# Personality Structure and Attachment in Bipolar Disorder

**DOI:** 10.3389/fpsyt.2020.00410

**Published:** 2020-05-11

**Authors:** Jolana Wagner-Skacel, Susanne Bengesser, Nina Dalkner, Sabrina Mörkl, Annamaria Painold, Carlo Hamm, René Pilz, Alexandra Rieger, Hans-Peter Kapfhammer, Michaela Hiebler-Ragger, Emanuel Jauk, Mary I. Butler, Eva Z. Reininghaus

**Affiliations:** ^1^Department of Psychiatry and Psychotherapeutic Medicine, Medical University of Graz (MUG), Graz, Austria; ^2^Department of Medical Psychology and Psychotherapy, MUG, Graz, Austria; ^3^Gruener Kreis Society, Center for Integrative Addiction Research, Johnsdorf, Austria; ^4^Institute of Psychology, University of Graz, Graz, Austria; ^5^Clinical Psychology and Behavioral Neuroscience, Technische Universität Dresden, Dresden, Germany; ^6^Department for Psychiatry and Neurobehavioral Science, University College Cork, Cork, Ireland

**Keywords:** bipolar disorder, personality structure, attachment, global symptom load, psychotherapy

## Abstract

**Background:**

An impairment of self and interpersonal functioning has an impact on coping strategies, regulation of affect and stress. Little is known so far about the impairment of personality functioning in patients with bipolar disorder (BD). The aim of this study is to assess the effects of personality structure and attachment in BD patients on the symptom burden.

**Methods:**

Forty-six patients with the diagnosis of BD were assessed by the 12-item Operationalized Psychodynamic Diagnosis Structure Questionnaire (OPD-SQS), the short version of Experience in Close Relationship-revised (ECR-R-D), and the Brief Symptom Inventory-18 (BSI 18) to determine the level of personality functioning, attachment patterns, and symptom load.

**Results:**

We observed positive correlations between personality difficulties, insecure attachment, and symptom load in patients with BD. A low level of structural integration and an insecure attachment style in patients with BD were accompanied by a significantly higher symptom load (*r* = 0.66, *p* ≤ 0.01). Interestingly, there were no significant differences in the structural integration (*T*(1.44) = −0.93, *p* = 0.357) and in the attachment style attachment related avoidance: (*T*(1,44) = 1.50, *p* = 0.140); attachment related anxiety (*T*(1,44) = −0.781, *p* = 0.439) of study participants with BD when compared to the normative value of the general population.

**Limitations:**

Our limitations are the small sample size of our group and the lack of a control group.

**Conclusion:**

In general, our results suggest that there is a link between personality structure and affective dynamics including depressive, anxiety, and somatization symptoms in BD. These findings underline the increasing importance of assessing personality structure and attachment for diagnosis and treatment planning of BD.

## Introduction

Bipolar disorder (BD) is a severe mood disorder, which is characterized by a cycling between the emotional extremes of mania and major depression. It has a lifetime prevalence of approximately 2% in the general population (1). There are conflicting study results about suicides in patients with BD, but it has been estimated that the risk of suicide in patients with BD is likely to be 20 to 30 times higher than in the general population (2). Lifetime suicide attempts are estimated to range from 25 to 50% (3).

Rates of relapse are high and many patients are not able to return to work — even when acute depressive or manic symptoms are absent — due to persisting subsyndromal symptoms and cognitive impairments (4, 5). A study undertaken in the US showed a high rate of unemployment of around 60% associated with BD (6). In line with this, it is known that individuals with BD report lower levels of functioning and well-being also in euthymic states. Functional domains, which are often impaired in BD, include personality structure, affective, cognitive, and self-regulatory resources as well as the quality of the self-other representation. These functional domains are important for the dynamic interplay of meaningful relationships (7). Following first onset of illness, only a third of individuals return to their former level of functioning within two years (5). Individuals with BD also have lifelong difficulties in relationships and the divorce rate is significantly higher in this group (8). Divorce rates are two to three times higher in adults with BD relative to the general population (9). A recent study demonstrated that both parents with BD and their intimate partners exhibit high levels of mental illness, maladaptive personality traits, and psychosocial difficulties, thus limiting their partners ability to provide support and stability (10). Personality structure represents the capacities for self and object recognition, regulation, communication, and attachment. Structural maladaptive developments result from the lack of fit between the child’s basic needs and the care options of the caregiver (11). These basic needs are tied to individual differences in personality; particularly those aspects of personality that are highly heritable and early-developing, commonly referred to as temperament (12).

Personality structure represents the capacities for self and object recognition, regulation, communication, and attachment. Impairment in personality functioning includes difficulties in interpersonal relations as well as self-regulation (13). A dimensional distinction of the degree of symptom severity seems to be important for indication and treatment planning. Numerous studies indicate that childhood trauma alters the clinical expression and course of BD contributing to earlier onset, increased risk for suicide attempts, rapid cycling, and co-morbid substance use (14). Individuals with BD and posttraumatic stress disorder experience high symptom burden and low quality of life (15). Emotional instability, novelty seeking, and anxiety are frequent in patients with BD, even when euthymic (16). Aspects of temperament and personality, such as affective instability and general anxiety, may be useful in defining specific subgroups of BD (17), a concept which is supported by findings of a genome wide association study of temperament, as a heritable stable personality factor, in BD (18). Furthermore, there is a need for characterization of temperament and personality traits in order to improve genetic risk factors for BD (19). Greenwood pronounced in a recent work creativity and BD as a model in which large doses of risk variants cause illness, but mild to moderate doses confer advantages. BD may thus be better conceptualized as a dimensional trait in positive temperament, personality, and cognitive abilities aspects of which may reflect a shared vulnerability with creativity (20).

In a recent paper, features of a vulnerable personality were found to be accompanied by cognitive deficits and emotional impairments in patients with BD (21). These links between cognition, emotion, and emotional regulation are of central importance for psychosocial functioning in both personal and vocational areas. Although patients with BD have difficulties in emotionality, emotion regulation, and emotion-relevant impulsivity (22), the literature investigating emotion and emotional regulation strategies in individuals with BD is sparse. Relevant aspects related to functionality and vulnerability within BD patients would be important for the development of novel treatment interventions. The connection of cognition and emotion in BD with the underlying structure of personality might be of importance when considering personality functioning and vulnerability to the disease, as personality difficulties lead to distinct impairments in self and interpersonal function (23).

### Personality Functioning

Personality functioning describes enduring maladaptive patterns of emotion, cognition, regulation, and behavior. Patients suffering with a personality disorder or impairment in personality functioning are significantly impaired in their psychosocial functioning, which includes difficulties in interpersonal relations as well as self-regulation. This leads to a high act of defiance on individual as social interactions. In the clinical setting these patients are experienced as difficult to treat (13). Due to the recent change of personality disorder classifications, in a dimensional or a composite categorical dimensional approach for personality pathology, the personality structure construct includes a broad range of personality facets and is very similar to the levels of personality functioning in DSM-5 (24). A growing number of studies confirmed the validity and reliability of the OPD structure questionnaire (OPD-SQ) in various clinical samples (25–27). The system of Operationalized Psychodynamic Diagnosis is an instrument with the effort to bring psychoanalysis and neuroscience together. The goal of the OPD Task Force was to broaden the ICD and DSM classifications to include fundamental psychodynamic dimensions, and at the same time to remain aspects of reliability and terminological precision apparent in ICD and DSM (28). The dimension of personality structure has been part of the psychoanalytic theory since Sigmund Freud presented his first structural model in 1900. Later on, he defined the short definition of the aim to be able to love and to work. These capacities can be regarded the precursors what we now call personality functioning. The functions of the ego helping an individual adjust and adapt to his or her reality (29). Personality functioning becomes visible in the shape of capacities or abilities of the self. The capacities contain for self and object recognition, regulation, communication, and attachment. We are involved in our environment; this arises from our perception and our memory. Our environment is bound to what we experience. We change in the light of the picture we make of ourselves.

The dimensional nature of personality, especially personality functioning along with a dimensional rating of the severity of personality dysfunction, has been accepted as highly important for indication and treatment planning in the new revisions of the two international classification systems (Diagnostic and Statistical Manual for Mental Disorders (DSM-5) and the upcoming International Classification of Diseases (ICD-11). The DSM-5 contains a Personality Functioning Scale with two domains “self” and “interpersonal” and four subdomains: “identity,” “self-direction,” “empathy,” and “intimacy” (30). The most important construct behind personality functioning is that of personality organization developed by Otto Kernberg (31). According to Kernberg, personality organization is reflected through three levels of functioning: coherence of identity, the maturity of defense mechanisms, and the ability to test reality (31). The dimensions of personality structure have been part of the psychoanalytic or psychodynamic theory and research since the first presentation of the structural model of Sigmund Freud (32). Kernberg developed the conceptualization of personality organization to differentiate between a healthy mature personality, a less severe, and a severe personality disorder (33). In the 1990s, the Operationalized Psychodynamic Diagnosis (OPD) completed the phenomenological classification system by psychodynamic dimensions (34). The OPD-2 axis IV aims to assess psychic structure (35) with the capacities and abilities for the recognition of self and other, emotional regulation, communication, and attachment.

In particular, the focus on domains beyond symptoms, such as global personality functioning has been accepted as highly important for indication and treatment planning (36). The OPD Structure Questionnaire (OPD-SQ) was developed to assess personality dysfunction in accordance with the OPD system (37). This instrument allows a time-efficient assessment of personality functioning that closely resembles actual patient experience and can be used for treatment planning and research.

### Personality Functioning in Bipolar Disorder

Patients with BD experience high levels of dependent negative life events (38) and engage in ineffective coping strategies to address stressful situations (39). Personal and social quality of life were directly predicted by depression symptoms and a diagnosis of BD and indirectly predicted by (hypo)maniac symptoms. Cognitive reserve seems to be a strong predictor of social well-being in patients with BD (40). In contrast, other patients are unable to work and struggle with interpersonal interactions. Social relationships and the capacity for attachment were found to be significantly compromised in individuals with BD (41). Nevertheless, research on personality functioning and attachment style in BD seems to be scarce and findings are contradictory. Patients with euthymia and depression in BD appear to be more dependent on other people than healthy controls (42).

### Attachment in Bipolar Disorder

Attachment in early childhood is extremely important for the formation of personality structure. Social relationships and attachment are the core developmental elements of human existence. Formed by the early family environment, our attachment system and processes have a lifelong impact (43). Attachment theory highlights the way through which normative processes support individual mental health or lead to pathology. In response to an inconsistent caregiver, anxious individuals develop hyperactivating strategies (44). These individuals often amplify distress and fail to regulate emotions (45). Many authors demonstrate the role of secure attachment leading to a physiological reactivity buffer to stress response compared to insecure attachment (46–48). The developmental aspects of attachment and social relationships have relevance for the early developmental epigenetic modification of gene expression which subsequently influences behavioral patterns (41). Furthermore, the association between attachment and social support may be highly relevant for the individual’s course of illness and life trajectory. Recent theoretical developments highlight the role of attachment from the therapeutic perspective. Specifically, epistemic trust (trust in the authenticity and personal relevance of interpersonally transmitted information) is thought to be an important factor for the therapeutic relationship (49). It helps the patient to change the rigidity that characterizes individuals with enduring personality difficulties (50). The re-learning of flexibility allows the patient to achieve changes in their understanding of social relationships, their own behavior and actions (49).

The association between the level of personality functioning, the adult attachment style, and the degree of psychiatric symptomatology in individuals with BD has not been thoroughly investigated. To our knowledge, this is the first pilot study using the combination of three contemporary tools to assess these variables.

The aim of our study is to investigate the differences in personality functioning and structure in individuals with BD in comparison to the personality functioning of the general healthy population using normative values (25). Individual differences in BD personality functions and structure might be critical for both the construction of new etiological models as well as the development of novel treatment strategies.

We formulated the following hypotheses for our study:

- Low structural personality integration (personality functioning) in patients with BD correlates with symptom perception.
- The BSI-18 score correlates negatively with OPD and OPD-subscales.
- The score of attachment correlates positively with the OPD score and negatively with the symptom-load (BSI-18 score).
- There is a significant difference between the structural integration and the attachment style of study participants with BD and normative values (25).

## Methods

### Participants and Recruitment

All participants were patients from our outpatient department for bipolar disorders and therefore in continuous clinical observation and care. All included participants were diagnosed by an experienced consultant psychiatrist with the Structured Clinical Interview for DSM-IV (SCID-I). Informed consent was obtained before each participant completed the online questionnaire.

The study measures included demographic variables (e.g. age, sex, education status, psychiatric diagnoses, medication, relationship status, current psychotherapy) as well as a standardized psychological test battery described in detail below. The data were acquired *via* the online-survey platform *LimeSurvey (www.limesurvey.org)*. Participants were included if they were aged between 18 and 65 years and completed all questionnaires. This study was approved by the local ethics committee of the Medical University of Graz (EK Nr: 24-123 ex 11/12). The assessment was carried out from September 2017 to January 2018.

### Psychometric Assessments

#### Level of Personality Functioning

We used the short version of the OPD Structure Questionnaire (OPD-SQS) for the assessment of personality functioning. The OPD-SQS is a viable screening instrument for supporting clinical decision making in treatment planning and therapy focus (25). The OPD-SQS consists of 12 Items with three subscales (self-perception, contact, relationship). The subscale “self-perception” combines aspects of self with structural skills of emotion regulation. The subscale “contact” combines interactional skills with aspects of self-uncertainty. The subscale “relationship” depicts the representation of relationship experiences and connections to expectations of new relationships. The score reaches from 0 (“highest structural level”) to 48 (“lowest structural level”). The internal consistencies range from α = 0.87 to 0.89 (25, 51).

### Attachment

We used the Relationship Structures questionnaire of the Experiences in Close Relationships-Revised (ECR-RS) that is designed to assess attachment dimensions in multiple contexts (52). The ECR-RS identifies four types of attachment including secure, preoccupied, detached, and fearful attachment, which correspond to the secure, ambivalent, avoidant, and disorganized attachment types described by Ainsworth (53). It contains attachment-related anxiety and avoidance features in four kinds of relationships: relationships with mother, father, romantic partners, and friends. The ECR-RS contains nine items assessing attachment in each of those four domains, therefore producing 36 items. Romantic attachment is associated with basic aspects of relationship functioning (52). Attachment-related anxiety is defined as involving a fear of interpersonal rejections, possessive relationship, low self-esteem, and distress when the partner is unresponsive. Attachment-related avoidance is defined as fear of dependence and interpersonal intimacy as well as the reluctance to self-disclose. High scores indicate insecure adult attachment styles while low scores can be viewed as having a secure adult attachment style (54). It employs a 7-point likert scale (1 = “absolutely disagree to 7 = “absolutely agree”). Cronbach’s α was 0.91 for attachment-related anxiety and 0.92 for attachment-related avoidance.

#### Global Symptom Load

Psychiatric symptoms and psychological distress were assessed with the Brief Symptom Inventory-18 (BSI 18) (55). The BSI comprises 18 items and is a short version of the Symptom-Checklist (SCL-90-R), which assesses psychological distress in the last seven days on three subscales (depression, anxiety, and somatization). The BSI-18 employs a five-point rating form ranging from 1 (absolutely not) to 5 (very strong). The subscales show an internal consistency with a Cronbach’s alpha of α = 0.79 for the sub-dimensions.

#### Manic Symptoms

Current (hypo-)manic symptoms were assessed with the Altman Self-Rating Mania Scale (ASRM) (56). The ASRM is a short five-item self-assessment questionnaire for assessing the presence and severity of manic and hypomanic symptoms. Manic subscale scores of greater than five on the ARSM resulted in values of 85.5% for sensitivity and 87.3% for specificity.

### Statistical Analyses

The assumption of a normal distribution was tested with the Kolmogorov Smirnov Test. Descriptive results of continuous variables are expressed as mean and standard deviation (SD) for Gaussian distributed variables. Data visualization was performed using GraphPad-Prism v5.

The Kolmogorov Smirnov Test showed that OPD contact, OPD sum, ECR-RS attachment-related avoidance were normally distributed. The other items (OPD self-perception, OPD contact, and OPD relationship) were not normally distributed.

All analyses were conducted in SPSS V23.0 (IBM, Paris, France).

To test our hypotheses, we used:

1. Correlation analysis (Pearson analysis in the case of normal distribution and Spearman correlation analysis in the case of non-normal distribution) to test the correlation between OPD-SQS scales, attachment, and global psychiatric symptom load measured with BSI-18.
2. One sample t-tests to compare OPD parameters from BD individuals with the well-defined normative values of healthy control persons according to the manual OPD-SQS (37).
3. One sample t-tests to compare attachment parameters (experiencing close relationships) from BD individuals with well-defined normative values of healthy controls according to the ECR-RD manual (52).

## Results

Forty-six individuals with BD were included in the study (25 males, 21 females). They were aged between 18 and 65 years (47.4 ± 15.4).

The individuals in our cohort divide into 23 patients with BD-1 disorder and 23 patients with BD-2 disorder. All participants were in euthymic states at the time of testing (BSI < 24, ASRM < 6). Demographic, clinical characteristics, and additional clinical information of the participants are given in **Table 1**.

**Table 1:** Demographic and clinical parameters of patients with Bipolar Disorder (N= 46).

Demographic and clinical parameters			
*Demographics*	*Mean (SD)*	*Min*	*Max*
Age (years)	47.4 (15.4)	18	65
*Sex*			
Males (n)	25		
Females (n)	21		
***Psychiatric symptoms***			
ASRM	0.50 (0.62)	0	2.60
BSI18 Depression	6.51 (5.46)	0	22
BSI18 Anxiety	4.30 (4.95)	0	21
BSI18 Somatization	4.11 (4.70)	0	18
BSI18 Sum	0.82 (0.71)	0	31
			
***Current Mood stabilizing medication***			
Lithium (%)	39.1%		
Anticonvulsants (%)	21.7%		
Antipsychotics (%)	30.4%		
***Clinical Parameters***			
Duration of the illness (years)	19.62 (13.94)		
Age of onset (years)	25.08 (10.32)		
Age of the first depression	25.71 (9.38)		
Age of the first mania	29.94 (12.37)		
Age of first diagnosis	38.35 (14.03)		
Number of suicide attempts	0.21 (0.42)		
Age when taking first lithium	38.35 (14.03)		
Cigarettes per day	0.52 (0.89)		
BMI	29.43 (6.41)		
Mental illness in the family (n)	31		
BD in the family history (n)	11		
Concurrent psychosis in history (n)	6		
Concurrent panic disorder (n)	6		
Concurrent personality disorder (n)	0		
Concurrent obsessive-compulsive disorder (n)	1		
Concurrent post-traumatic stress disorder (n)	1		
Concurrent migraine (n)	7		
Concurrent diabetes mellitus (n)	0		
Concurrent hypertension (n)	6		
Concurrent heart disease (n)	5		
Concurrent vascular disease (n)	7		
Concurrent cardiovascular disease (n)	8		
Concurrent pulmonary disease (n)	12		
Concurrent gastrointestinal disease (n)	5		
Concurrent neurological disease (n)	8		
Concurrent endocrinological disease (n)	9		

BSI 18, Brief Symptom Inventory (total value of each scale is 24); ASRM, Altman Self-Rating Mania Scale (0–20 total value; Cut Off >6 maniac symptoms or hypomanic condition).

The educational level of the participants was as follows: patients with basic education (4.3%), secondary education (15.2%), secondary upper education (39.1%), and university education (32.6%). The remaining participants did not complete this section of the questionnaire (8.7%). The patient population consisted of 65.2% participants in a relationship and 34.8% currently without a partner.

The patients were all in our specialized outpatient clinic for bipolar disorders with a treatment plan consisting of medication, psychoeducation, and psychotherapy (upon need). 73.9% of the participants had frequent psychotherapy sessions. At time of testing, all participants were on medication including mood stabilizers (lithium, atypical antipsychotics, and anticonvulsants) and antidepressants. **Table 1** is giving an overview regarding mood stabilizers. 91.7% of patients were taking mood stabilizers at time of testing.

**Figure 1:**
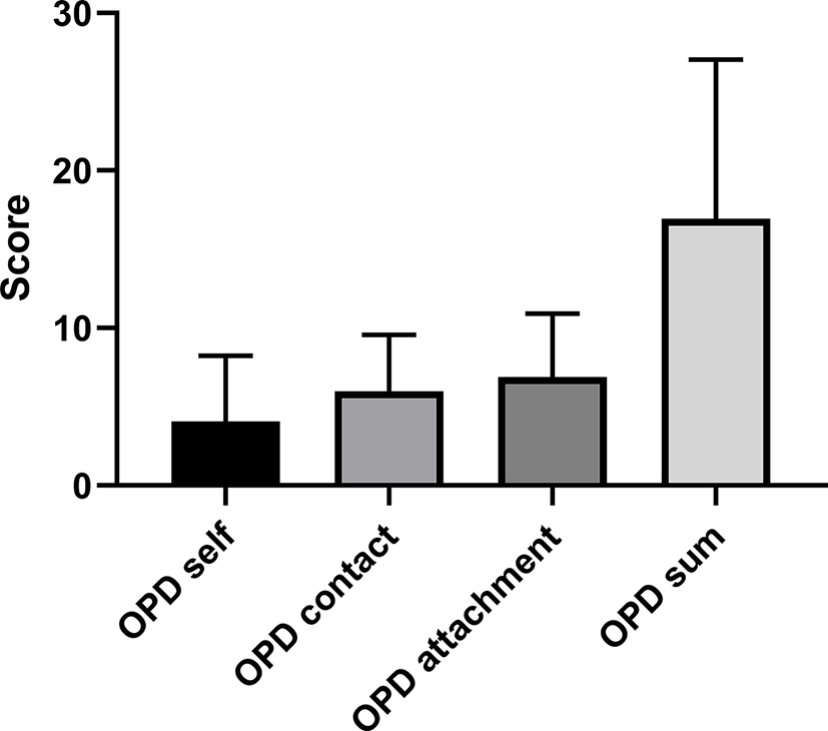
Mean and standard deviation of the OPD sum score and sub-scores in bipolar disorder (N= 46).

**Figure 2:**
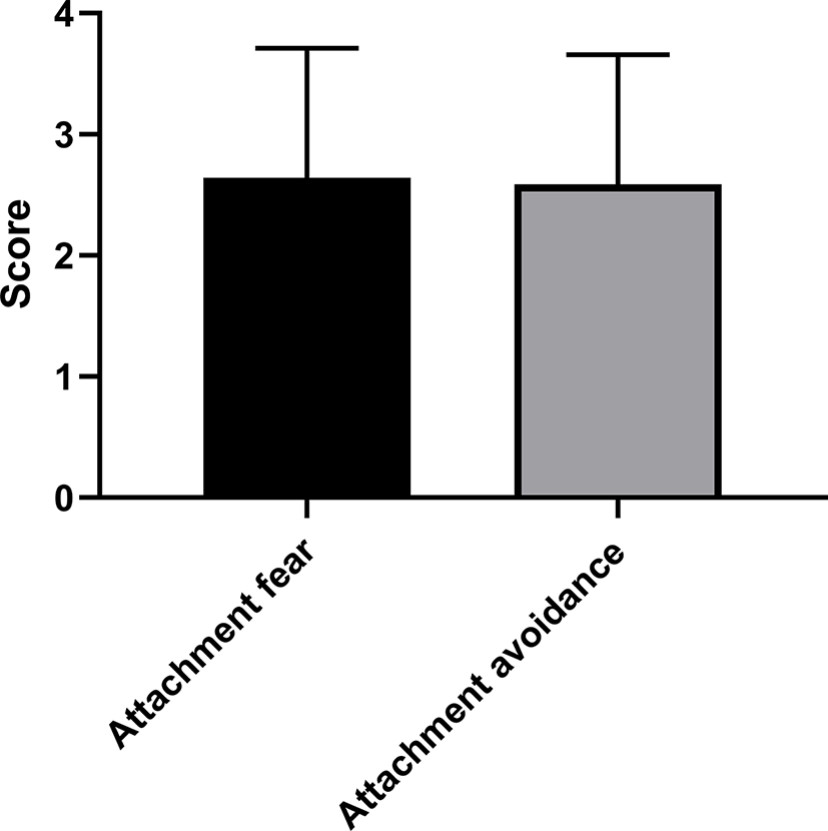
Mean and standard deviation of ECR-RS in bipolar disorder (N= 46).

**Figure 1** shows the mean and standard deviation of the OPD sum score and sub-scores. **Figure 2** depicts the mean and standard deviation of *ECR-RS* (attachment-related anxiety and avoidance). **Table 2** shows means and standard deviations of the subscales self-perception, contact, and relationship of OPD-SQS and the ECR-RS scales “attachment-related anxiety” and “attachment-related avoidance.”

**Table 2:** OPD scores and attachment styles (N= 46).

OPD scores and attachment styles			
	Mean (SD)	Min	Max
***OPD-SQ***			
Self-perception	4.07 (4.16)	0	15
Contact	5.98 (3.58)	0	15
Relationship	6.89 (4.01)	0	15
***ECR-RS***			
Attachment-related anxiety	2.64 (1.07)	1.0	5.28
Attachment-related avoidance	2.58 (1.06)	1.0	5.22

OPD-SQS, Operationalized Psychodynamic Diagnosis OPD self questionnaire short version; ECR-RS, Relationship Structures questionnaire of the Experiences in Close Relationships-Revisited.

### One-Sample t-Tests

There was no significant difference in the parameter “attachment related avoidance” between study participants with BD and the normative value of the *ECR-RS* manual (*T*(1,44) = 1.50, *p* = 0.140).

There was no significant difference in “attachment-related anxiety” between study participants with BD and the normative value of the general population (*T*(1,44) = −0.781, *p*= 0.439). The mean of the “attachment-related anxiety” score was 2.64 ± 1.07.

There was no significant difference in the parameters of the OPD sum score of study participants with BD compared to the normative value of the general population (*T*(1.44) = −0.93, *p* = 0.357)

### Correlation Analysis

#### Association of OPD Scales, Attachment, and Psychiatric Symptoms in Individuals With BD

OPD self-perception correlated positively with attachment-related anxiety (*r* = 0.55, *p* < 0.001), attachment-related avoidance (*r* = 0.36, *p* = 0.017), and BSI scales (see **Table 3**).

**Table 3:** Relationship of OPD and Attachment scales (ECR-RS) with psychiatric symptoms (BSI 18).

	BSI18 Depression	BSI18 Anxiety	BSI18 Somatization	BSI18 Sum
OPD Sum	0.68***	0.60***	0.37*	0.66***
OPD Self-perception	0.63***	0.51***	0.38*	0.60***
OPD Contact	0.58***	0.58***	0.26	0.57***
OPD Relationship	0.49**	0.42**	0.29	0.48***
ECR-RS Attachment-related avoidance	0.42**	0.35*	0.43**	0.47**
ECR-RS Attachment-related anxiety	0.47**	0.27	0.25	0.40**

BSI18, Brief Symptom Inventory; OPD, Operationalized Psychodynamic Diagnosis OPD self questionnaire short version; ECR-RS, Relationship Structures questionnaire of the Experiences in Close Relationships-Revisited; ***=p ≤ 0.001, **=p ≤ 0.01,*=p ≤ 0.05.

OPD contact correlated significantly with attachment-related anxiety (*r* = 0.46, *p* = 0.002) and attachment-related avoidance (*r* = 0.34, *p* = 0.021). OPD relationship correlated significantly with attachment-related anxiety (*r* = 0.31, *p* = 0.038) and BSI scales (see **Table 3**), but not with attachment-related avoidance (*r* = 0.28, *p* = 0.059). OPD sum correlated significantly with attachment-related anxiety (*r* = 0.50, *p* < 0.001), attachment-related avoidance (*r* = 0.388, *p* = 0.008), and BSI scales (see **Table 3**).

## Discussion

The aim of our study was to assess the impact of personality function and attachment in individuals with BD on their symptom load. Our data demonstrate that individuals with BD and impaired personality functioning show more psychological distress and higher symptom perception. Similarly, individuals with BD and an insecure attachment style show more psychological stress and higher symptom load.

The possibility of differences in personality structure between BD patients and the common population is derived from clinical experience and psychodynamic theory. According to psychodynamic theory, a patient with a lower structural integration shows symptom intensification and a lower therapy response. The scale “self-perception” combines aspects of the self with structural skills of emotional regulation (51). In our participants, we detected a high correlation with somatization, anxiety, and depression. Remarkably, there is a close relationship between self-perception and negative affect. Moreover, patients with a low self-perception, low affect differentiation, and a low sense of identity may be more anxious and more possessive. These findings are consistent with the significant correlation between self-perception and insecure attachment style found in our study.

The scale “contact” combines interactional skills with aspects of self-uncertainty. These aspects lead to a more pronounced experience of stress in patients with BD. The pattern “contact of personality functioning” seems to reflect a high ratio of anxiety and depression and a lower prevalence of somatization in the BD sample. One might assume that the pattern contact shows a higher impairment in the realms of relational functioning as well as coping with stress. This could illustrate an increased need for psychotherapeutic support for patients with BD and concomitant impairment in personality functioning. In this context, Hemminghoffen and colleagues have demonstrated that psychotherapeutic attitude towards BD patients should include a more active role of the therapist. Moreover, the way patients with BD relate to other people is very important for treatment adherence and prevention of relapse (57, 58).

The scale ‘relationship’ forms the representations of relational experiences connected with appropriate expectations of new relationships (25). This scale correlates highly with depression and anxiety in our BD participants. The importance of relationships helps us to understand patient health behavior and patient–provider relationships. Attachment theory has been used to explain individual differences in coping behavior and self-treatment and to understand the behavior of patients with chronic illness (43). Integrative approaches to psychotherapy demonstrate the significance of the personal recognition of the patient by the therapist. This process is fundamental as it allows the patient to acquire new behavior, thus creating the openness to follow further therapeutic suggestions (59). Patients with BD and personality dysfunction often struggle in therapy because their disorder serves to undermine their capacity to benefit from the therapeutic process. Both individual and group therapy can serve to activate the attachment system. Our data demonstrate the importance of recognizing structural dysfunction in order to highlight the potential benefit of structure-based and attachment-oriented therapy for patients with BD. These therapies allow patients to benefit from social interventions and to update and build on knowledge about the self and others in social situations. It further underlines the capacity of the therapeutic relationship to create a potential for learning about oneself and others.

Attachment-related anxiety, defined as a fear of interpersonal rejection associated with a need for attention and a tendency to show stress reactions if the partner is not available, was significantly associated with depression in our population.

Attachment-related avoidance is defined as the rejection of dependency and interpersonal proximity (associated with strong self-absorption and a lack of self-disclosure). In our BD participants, this subscale showed a strong correlation with somatization, anxiety, and depression. Attachment-related avoidance could therefore be associated with more distress owing to a stronger self-absorption and a lack of self-disclosure (60).

Individuals with high scores on one or both scales have an insecure attachment with an impairment of interpersonal proximity and distance as well as an impairment of the regulation of affect and stress. These patients with BD could have inferior protective factors during times of distress, anxiety, and illness.

Researchers have suggested a significant association between insecure attachment and mood disorders (61–64). In a few studies in BD (65) researchers have observed a higher prevalence of insecure attachment, especially the anxious type and noted some influence on the stage of the disorder.

However, in our BD participants there was no significant difference in the parameter attachment-related anxiety compared to the normative value of the general population. This leads us to the limitations of our study. One limitation was the online survey which meant that there was no personal contact with the study participants. Another limitation is the small sample size of our group. This reduces the generalizability of our results regarding personality functioning in BD. Other issues worth considering are that the attachment and personality structure parameters did not differ compared to the normative values. This could be due to several factors. There is the possibility of a selection bias, in that it is possible that more well -integrated patients in the outpatient care were more prone to fill out our email survey. In addition, medication and psychotherapy could have influenced the outcome.

Interestingly, there were no differences in personality structure between patients with BD and the normal population. Furthermore, there were no differences in the attachment style between individuals with BD and the normal population. Reasons for not finding differences to normative values of the general population could be rooted in the multifactorial nature of BD and the predominant role of genetics and epigenetic factors (66) as in the pre-existing psychotherapeutic treatment.

The lack of differences between the BD patients and the normative value could be because the patients were receiving psychopharmacological and psychotherapeutic treatment at the time of testing. We sent the survey to 80 patients of our outpatient clinic. Only 46 persons (23 patients with BD type 1 and 23 patients with BD type 2) answered the questionnaires. The type of illness (bipolar I disorder vs. bipolar II disorder) could also have had an impact on personality functioning. However, our subgroups of 23 patients each are relatively small, and therefore we cannot draw reliable conclusions. It could be possible that the patients who answered were responding better to treatment and had a better relationship to the therapist (with more trust and better compliance). This better therapy response could have also been due a relatively long duration of illness (with a mean duration of illness of 19.62 years). Another important limitation is that some patients had co-diagnosis such as anxiety disorders (see **Table 1**), however, none of the patients had a diagnosed personality disorder.

The pathophysiological pathways of BD are different to the etiological pathways of a personality disorder. The cognitive and affective dysregulation of patients with BD could be reinforced through an impairment in personality functioning. Additionally, there are different mutually influencing biological, psychodynamic, and learning theory pathways involved in the development of these diseases. In light of this, it may be necessary to extend the existing diagnosis system through this additional phenomenological consideration.

Our limitations are the small sample size of our group and the lack of a control group. Nevertheless, we used the well described normative data from Ehrenthal et al. (25). He used the original sample of the OPD-SQ (N=1110) evaluation for the short form OPD-SQS with 204 inpatients, 172 outpatients, and 734 control subjects (without ongoing psychotherapy). This original sample was randomly halved in a construction sample for the evaluation of OPD-SFK and a review sample to verify the short version in terms of factor structure and validity. After developing a preliminary 12-items version in one sub-sample, they used confirmatory factor-analysis in the second subsample as well as an independent sample to test the factor structure. The internal consistency was calculated as a measure of reliability. The OPD-SFQ is a viable screening instrument for supporting clinical decision making in therapy planning (25). Many patients have characteristics of a personality disorder in their experience and their styles of interaction, but they do not reach the threshold of the full image of a personality disorder (67).

A major limitation of our study is the lack of a specific depression inventory such as the BDI. We measured the severity of depression with the BSI-18, a short form of the SCL-R-90 (68), which also measures somatization and anxiety. All our participants had a sum score <24. Only one patient reached a BSI-18 sum score of 22. For the mood state of mania, we have used the Altman Self Rating Mania Scale (ASRM) with a cut off under 6. None of our participants fulfilled the criteria for mania at the time of testing.

The assessment of personality function and attachment makes it possible to identify relevant intrapsychic and interpersonal conflicts and problems of personality structure in BD patients (69). In future it should be possible to diagnose problems in structure to optimize the psychotherapeutic relationship. This may be of particular value when making decisions about psychotherapeutic treatment. The findings here suggest that the psychotherapeutic attitude should show a more active role for the therapist and a greater integration of a interactive dialogue in the outpatient care and clinical setting. The focus should lie on treatment continuity with frequent supportive consultations and an interactive attitude of the therapist.

The patients with an impairment of personality functioning should receive a modified psychotherapeutic intervention. These patients need more information about their illness, their medication in addition to receive more information about their emotional regulation. They have a need of a frequent setting with a more active role of the therapist or doctor. The self-awareness of patients with an impairment in personality functioning should be improved in strategies and skills for the emotional regulation, the regulation of relationships and a regard on healthy living. Personality functioning may be especially important in chronic diseases such as bipolar disorder, that demand a high level of compliance and lifestyle change (13). The association between severe psychiatric disorders and metabolic syndrome goes along with a reduced life expectancy. Characterizing somatic comorbidities in patients with severe psychiatric disorders can lead to a better understanding in possible bidirectional etiologic factors (70). It is reasonable that alterations in personality functioning on a subsyndromal level outside of personality disorder could have an impact on the subject’s impairment in regulatory capacities. So, levels of personality functioning could be predictive for the patient’s compliance and lifestyle change in favor of a better health management. Characterizing the personality structure in patients with BD could help to predict a better symptom management and personalized therapy treatment with modulating the therapy in a more instructional therapy. The influence of personality functioning on compliance and somatic comorbidities with metabolic syndrome should be part in future research.

## Conclusion

An impairment of personality functioning and attachment in individuals with BD leads to more psychological distress. This should be taken into consideration when diagnosing and exploring treatment options for patients with BD. Assessment and characterization of personality and attachment styles may be of particular value in identifying individuals who may respond to certain forms of psychotherapeutic treatment.

## Data Availability Statement

The datasets generated for this study are available on request to the corresponding author.

## Ethics Statement

The studies involving human participants were reviewed and approved by ethics committee of the Medical University of Graz, Protocol Number: 24-123 ex 11/12. The patients/participants provided their written informed consent to participate in this study.

## Author Contributions

JW-S has designed the study, written the first draft, was responsible for the study conception, coordination, and publication of data. SB was involved in the conception of the study and responsible for patient recruitment and testing. In addition, she supervised and guided us through the whole process of analysis and publication. ER was responsible for study conception and design, coordination, funding, and drafting/publication of data. ND, as our responsible psychologist, managed patient schedules and appointments, and supervising the testing. EJ was responsible for the scientific personality parameters in this project. SM was involved in the conception of the study and did revision for important intellectual content. MH-R was responsible for the clinical, scientific work up. She helped with data analysis. CH was responsible for the clinical, scientific work. H-PK supervised us during the whole study procedure. AR was involved in the conception of the study. MB was responsible for the proof reading as a native speaker. RP was involved in developing the study protocol and testing. AP was involved in the proof reading and submission.

## Funding

This work's open access fees were supported by the government of Styria.

## Conflict of Interest

The authors declare that the research was conducted in the absence of any commercial or financial relationships that could be construed as a potential conflict of interest.
